# 

**Published:** 1870-02

**Authors:** 


### CONTENTS:

ORIGINAL COMMUNICATIONS:

ART. I. On the Origin, History and Treatment of Trichina Spiralis, - - - - - 65

" II. Modus Operandi and Domain of the Turkish Bath. By M. P. Hanson, M.D., - 82

" III. Puerperal Convulsions. By J. Little, M.D., - - 85

" IV. Cases of Syphilis. By F. D. Norcom, M.D., - - - 92

" V. Microscopic Investigations in Cases of Sterility. By B. H. Cheney, M.D., - - 101

" VI. Report of a Case of Typhoid Fever, showing Range of Temperature from Beginning to the End of the Case. By T. C. Murphy, M.D., 101

" VII. Abscence of the Ductus Communis Choledochus. By I. N. Danforth, M.D., - 110

FOREIGN CORRESPONDENCE:

Letters from Paris. By A. W. Bosworth, - - - - - - - 111

EDITOR'S BOOK TABLE, - - - - 119

EDITORIAL:

Rush Medical College. Names of Gradates, - - - - - - - 121

Alumni Meeting, - - - - - 123

Diplomas for Sale, - - - - 123

Ancient Sanitary Regulations, - - 126

Unique Diagnosis, - - - - - 126

